# Do we need to reconsider the CMAM admission and discharge criteria?; an analysis of CMAM data in South Sudan

**DOI:** 10.1186/s12889-020-08657-x

**Published:** 2020-04-16

**Authors:** Eunyong Ahn, Cyprian Ouma, Mesfin Loha, Asrat Dibaba, Wendy Dyment, Jaekwang Kim, Nam Seon Beck, Taesung Park

**Affiliations:** 1 Korea Basic Science Institute, Seoul, Korea; 2 Research Institute of Basic Science, Seoul National University, Seoul, Korea; 3 World Food Programme, Regional Bureau-Nairobi, Nairobi, Kenya; 4 East Africa Regional Office, World Vision, Nairobi, Kenya; 5 Medair, Ecublens, Ecublens, Switzerland; 6 Department of Statistics, Iowa State University, Ames, USA; 7 Department of Statistics, Seoul National University, Seoul, Korea; 8 Interdisciplinary program in Bioinofrmatics, Seoul National University, Seoul, Korea

**Keywords:** Mid upper arm circumferences (MUAC), Severe acute malnutrition (SAM)

## Abstract

**Background:**

Weight-for-height Z-score (WHZ) and Mid Upper Arm Circumference (MUAC) are both commonly used as acute malnutrition screening criteria. However, there exists disparity between the groups identified as malnourished by them. Thus, here we aim to investigate the clinical features and linkage with chronicity of the acute malnutrition cases identified by either WHZ or MUAC. Besides, there exists evidence indicating that fat restoration is disproportionately rapid compared to that of muscle gain in hospitalized malnourished children but related research at community level is lacking. In this study we suggest proxy measure to inspect body composition restoration responding to malnutrition management among the malnourished children.

**Methods:**

The data of this study is from World Vision South Sudan’s emergency nutrition program from 2006 to 2012 (4443 children) and the nutrition survey conducted in 2014 (3367 children). The study investigated clinical presentations of each type of severe acute malnutrition (SAM) by WHZ (SAM-WHZ) or MUAC (SAM-MUAC), and analysed correlation between each malnutrition and chronic malnutrition. Furthermore, we explored the pattern of body composition restoration during the recovery phase by comparing the relative velocity of MUAC^3^ with that of weight gain.

**Results:**

As acutely malnourished children identified by MUAC more often share clinical features related to chronic malnutrition and minimal overlapping with malnourished children by WHZ, Therefore, MUAC only screening in the nutrition program would result in delayed identification of the malnourished children.

**Conclusions:**

The relative velocity of MUAC^3^ gain was suggested as a proxy measure for volume increase, and it was more prominent than that of weight gain among the children with SAM by WHZ and MUAC over all the restoring period. Based on this we made a conjecture about dominant fat mass gain over the period of CMAM program. Also, considering initial weight gain could be ascribed to fat mass increase, the current discharge criteria would leave the malnourished children at risk of mortality even after treatment due to limited restoration of muscle mass. Given this, further research should be followed including assessment of body composition for evidence to recapitulate and reconsider the current admission and discharge criteria for CMAM program.

## Background

Forty nine million children of age 5 years or less were reported wasted in 2018 [1]. Though malnourishment among children aged 5 years or less has decreased by 10% since 1990, the prevalence of wasting was estimated at nearly 7.3% in 2018 [1]. Additionally, UNICEF reports that more than 1.5 million children mortality occurred due to severe acute malnutrition every year [2] and 3.5 million children of age 5 years or less with moderate acute malnutrition succumb to death in 2005 [3]. Acute malnutrition is defined by a decrease of two standard deviations (SD) below the WHZ (Weight for Height Z score) [4] while chronic malnutrition, described as stunting, is defined by a decrease of two SD below the HAZ (Height for Age Z score) [5].

For children aged between 6 and 59 months, the World Health Organization (WHO) and the United Nations Children’s Fund (UNICEF) have advised to use both WHZ and Mid Upper Arm Circumference (MUAC) as well as bipedal oedema for identification of children with Acute Malnutrition [6, 7]. However, MUAC tends to replace WHZ and to be considered as a single indicator in the community together with bipedal oedema for malnutrition screening [7, 8]. This trend is rationalized by MUAC’s superiority in predicting mortality and has other benefits such as simplicity and accuracy of the measurement, easy training and its attribute of being less affected by dehydration [6, 7, 9–14]. Furthermore, the prognostic accuracy of MUAC to predict mortality is not inferior to that of combined screening by both MUAC and WHZ or even MUAC z-score for age [14, 15].

However, neither MUAC nor WHZ on its own screening is able to capture a significant portion of children who succumbed to malnutrition [14]. It is also recognized that each screening measure is poorly related [14, 16], and there still exists uncertainty about the characteristics of the two malnourished types by either MUAC or WHZ [17].

Furthermore, chronic irreversible sequelae including failure of intellectual development and stunting are not properly reflected in the current community screening guideline. There is some evidence showing that SAM-WHZ represents a potential stunting risk better than SAM-MUAC [18–21], and several studies proved that if the children succeed in timely weight gain, following catch up growth could be observed after the treatment from nutrition program [22–26]. In other words, unless appropriate management takes place in time, catch-up growth hardly occurs afterward [27]. If we use MUAC as the single modality for community screening of acute malnutrition, we might lose opportunity for catch-up growth for those who are excluded by MUAC criterion but still malnourished by WHZ criterion.

For discharge from Community Management of Acute Malnutrition (CMAM) program, WHO recommends to use the anthropometric criteria for admission [28]. Severely wasted children lose their muscle mass almost a half or two third of the amount expected for their height, which would indicate that muscle mass depletion is more often in the children with SAM [29, 30]. A few studies showed initial weight gain among the malnourished children who were hospitalized was primarily due to rapid fat mass increase rather than muscle mass gain [31–33]. If these are also valid for the malnourished children enrolled in CMAM program, which is designed to identify early stage of malnourished cases and provide timely management in communities before developing complication to limit cases demanding hospitalization, simple recovery of anthropometric measures does not necessarily mean full restoration of each body composition. Furthermore, this would lead to avoidable recurrence and/or mortality after discharge from CMAM program considering that low muscle mass is the main mortality risk [33]. Several studies revealed unacceptable high mortality and recurrence rate after discharge from CMAM program [34–37].

Here, this study aimed to characterize the clinical features of the malnourished children by diagnostic modalities; estimate correlation of each type of acute malnutrition with chronic malnutrition; and explore restoration pattern of body composition responding to management by analysing program and survey data.

## Methods

### World vision has been implementing CMAM

program in South Sudan since 2006 to care for malnourished children aged 0–59 months. All malnourished children in the community identified by using the criteria (MUAC < 125 mm, WHZ < − 2, or edema) were put in the CMAM program, and cared until both MUAC and WHZ reached normal. Except the cases with edema, the CMAM program data from 2006 to 2012 have been collected regularly for analysis resulting in a total of 4443 cases in hardcopy. The data were transferred to spread sheets for data pre-processing and analysis. Additionally, the nutrition survey data from the community were collected in spread sheets through a series of nutrition surveys (Standardized Monitoring and Assessment of Relief and Transitions, SMART [38]) from 2013 to 2014 covering the program target communities.

MUAC was measured using a UNICEF non-stretch tape to the nearest 1 mm. Weight was measured with the child wearing no clothes or light clothes to the 0.1 kg by a Salter analog scale while length/height was measured to the nearest 0.1 cm with a Holtain infantometer. Age was estimated to the nearest month by reviewing birth certificates, asking the date of birth or using event calendar. The anthropometric data was transformed to Z-scores by the WHO’s standard [6, 39].

For the CMAM program data, originally paper based records are transferred to Microsoft Excel sheets. While trainee are typing hand written records to soft copies, some of the records are either duplicated or incorrectly written. In order to deal with potential measurement error, we perform the quality control as Table 1. A total of 3479 data from the program and 3358 from the community nutrition survey were secured for analysis after data pre-processing and parsing process.

**Table 1:** Data parsing and cleaning for the program data

Description		Number of records
Raw data	15 Excel files	4443
Exclude the overlapped records	Based on name, weight, and height	3603
First quality control	6 exclusion criteria ^a^	3504
Second quality control	4 exclusion criteria ^b^	3479

^a^ Exclusion criteria for the first quality control; (1) max (height) - min (height)> 5cm, (2) max (weight) - min (weight)> 5kg, (3) max (height)> 120cm (~mean+3SD of the data), (4) min (height)< 45cm(~mean-3SD of the data), (5) max (weight)> 20kg (~mean+3SD of the data), (6) min (weight)< 3kg(~mean-3SD of the data). ^b^ Exclusion criteria for the second QC; (1) WFH> 130(~mean+3SD of the data), (2) WFH< 50(~mean-3SD of the data), (3) z-score > 3, (4) z-score < -7

**Fig. 1:**
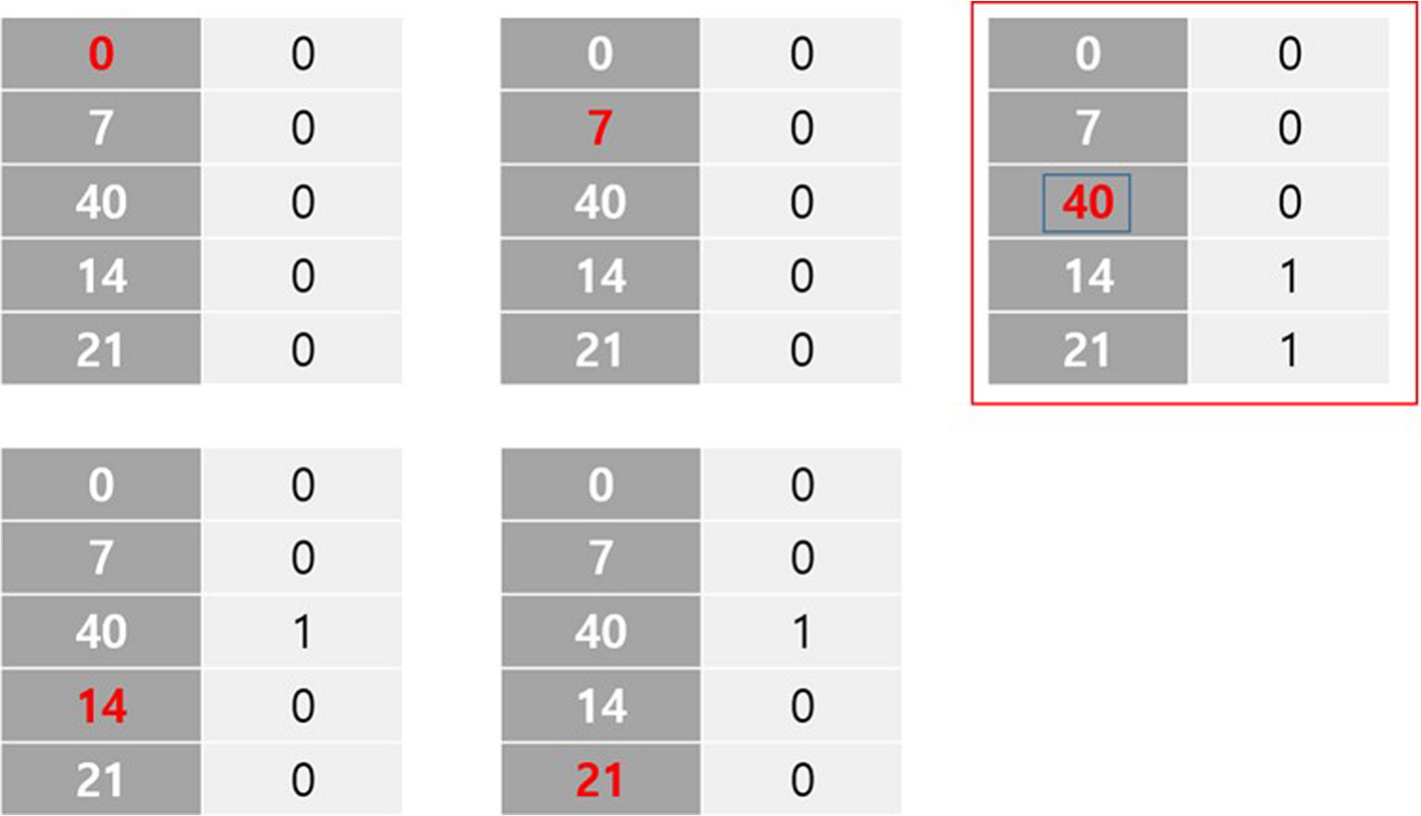
The methodology to replace decreasing dates to NA. When there is a decreasing dates in a sequence, we replace the date with the biggest penalty. To calculate the penalties, for each date, if there is a bigger (smaller) day before (after) the chosen date, penalty was given

Quality control for the recorded dates in program data is performed to investigate children’s recovery patterns. Specifically, decreasing dates are erased from the data. To define wrong dates written by mistake, we defined simple methodology (Fig. 1). Detected 157 wrong dates with the biggest penalties are replaced with N/A. After the correction, still records from 31 children include decreasing dates, and these are leaved out from the data analysis. Besides, if a child was absent for three consecutive visits, next visit is considered as a new episode for the analysis. Finally, we used repeatedly measured clinical features in 3977 episodes from 3448 children from South Sudan nutrition program data.

The researchers characterize acute malnutrition by investigating clinical presentations of each malnutrition by MUAC or WHZ including age and sex at presentation, and correlation of each type of acute malnutrition with chronic malnutrition, stunting.

As long as lean body mass (LBM) and fat mass restore homogeneously with same speed, the relative velocity weight and volume gain remains same throughout the recovery phase. However in case where fat gain is predominant, relative velocity of volume gain is faster than that of weight gain as fat is less dense than lean body by 18% [37, 40]. As MUAC measures physical length, MUAC^3^ is possibly considered as a proxy to body volume, while body weight reflects body mass. Here we hypothesized that the relative rate of fat and muscle gain could be estimated by comparing relative rate of MUAC^3^ and weight gain. On comparing the increase in measurements, data from all admission cases passed quality control was analysed without any restriction on outcomes. We used sliding window (30 day width) to estimate the daily relative increase because children’s visits are not exactly every one week after first time and estimated points are smoothen to roughly draw the trend line.

## Results

### Clinical features of each type of acute malnutrition

**Fig. 2:**
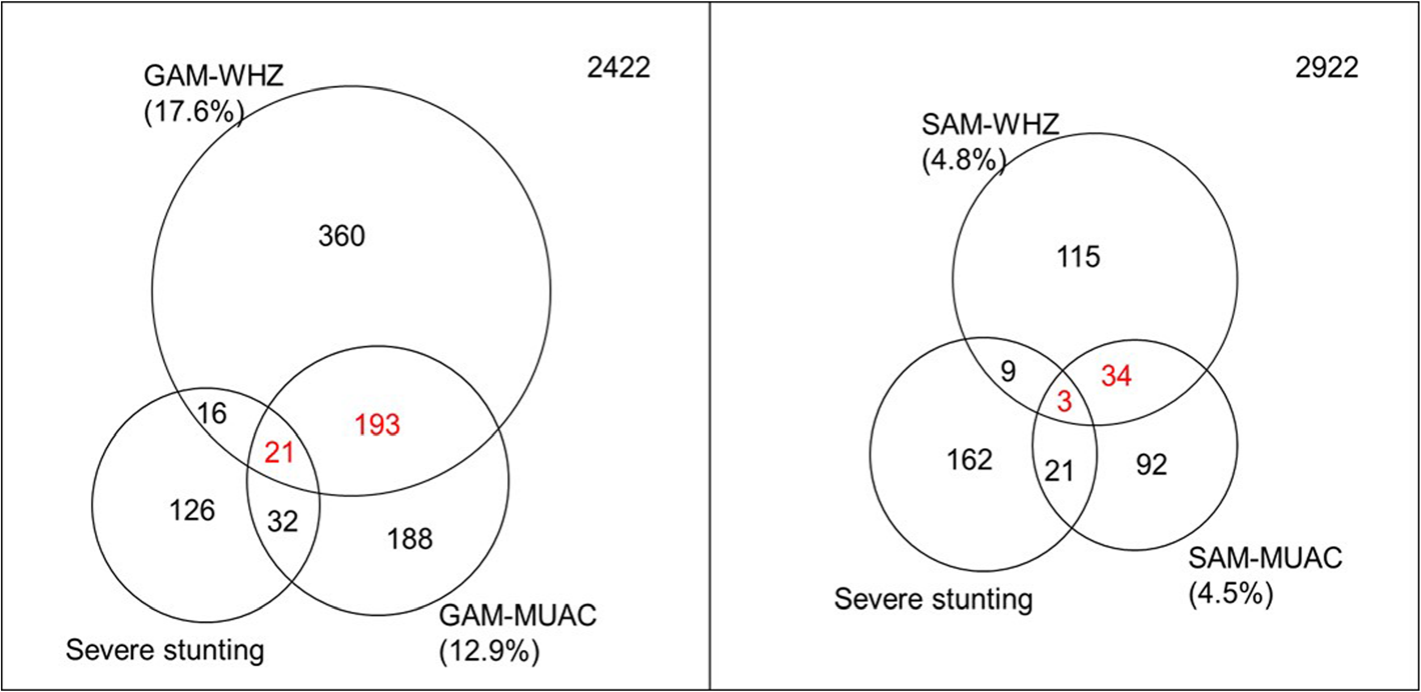
The Venn diagram of the children with acute malnutrition by MUAC and WHZ from the community data. WHZ GAM, global acute malnutrition by WHZ (<− 2); MUAC GAM, Global Acute Malnutrition by MUAC (< 125 mm); WHZ SAM, Severe Acute Malnutrition by WHZ (<− 3); MUAC SAM, Severe Acute Malnutrition by MUAC (< 115 mm); Severe stunting, HAZ (<− 3)

The Venn diagram (Fig. 2) of the community data shows that the prevalence of SAM-MUAC is similar to that of SAM-WHZ (4.8 vs. 4.5%, respectively), which is the same for Global Acute Malnutrition (GAM, defined as MUAC < 125 mm or WHZ < − 2) (12.9% ± 1.3% vs. 17.6% ± 1.1% respectively). The Venn diagram also indicates that only 13.5% (37 out of 274) and 26.4% (214 out of 810) are overlapped between the two SAMs’, and two GAMs’ respectively.

The median age for children admitted by SAM-WHZ to the malnutrition program is 29 months, which is greater than that of children admitted by SAM-MUAC, 18 months. There is no predilection of sex for each type of SAM and GAM both in program (*p* = 0.2483) and community data (*p* = 0.6771) (Table 2. A). However, more malnourished girls were identified by MUAC than WHZ (*p* = 0.01905) (Table 2. B).

**Table 2:** The clinical characteristics of each type of SAM and GAM

**A. Sex distribution for SAM and GAM both in program and community survey**						
			Program		Community Survey	
SAM	Male		1063 (30.6%)		144 (4.3%)	
	Female		936 (26.9%)		153 (4.6%)	
GAM	Male		1527 (43.9%)		419 (12.5%)	
	Female		1440 (41.4%)		417 (12.4%)	
Total			3479		3358	
**B. Association between SAM-MUAC or SAM-WHZ and SEX in community survey data**						
	**SAM-MUAC**			**SAM-WHZ**		
	**Normal**	**SAM**		**Normal**	**SAM**	
**Male**	**1641**	**75 (4.4%)**	**Male**	**1643**	**73 (4.3%)**	
**Female**	**1548**	**103 (6.2%)**	**Female**	**1596**	**55 (3.3%)**	
**C. The Proportion and Odd Ratio of each type of SAM by height**						
	WHZ Regardless of MUAC		WHZ Normal by MUAC		MUAC Regardless of WHZ	
	< −3 (SAM)	≥ − 3	< − 3 (SAM)	≥ − 3	< 115 mm	≥ 115 mm
Children with height falling on upper quartile	48 (5.7%)	793	41 (5.0%)	772	28 (3.3%)	813
Children with height falling on lower quartile	30 (3.6%)	810	18 (2.3%)	758	64 (7.6%)	776
Odd Ratio and *P* value	OR 1.63 (p = 0.049)		OR 2.24 (p = 0.006)		OR 0.42 (p = 0.0001)	
**D. The Proportion and Odd Ratio of each type of SAM by height**						
	MUAC			WHZ		
	< 115 mm		≥ 115 mm	< −3 (SAM)	≥ −3	
HAZ < − 3	24 (12.3%)		171	12 (6.2%)	183	
HAZ ≥ − 3	126 (4.0%)		3037	149 (4.7%)	3014	
Χ^2^ statistics (*P*-values)	27.9053 (1.274 × 10^−7^)			0.5517 (0.4576)		
**E. The duration of recovery to weight and MUAC gain for each type of the SAM cases**						
① Number of Days to 15% weight gain						
	No. of Records	Events	Median		0.95 LCL^a^	0.95 UCL^b^
Z < −3 (WFH)	813	433 (53.3%)	46		42	49
MUAC < 115 mm	810	383 (47.3%)	49		42	52
② Number of Days to 5% MUAC						
Z < − 3 (WFH)	818	438 (53.5%)	35		35	42
MUAC < 115 mm	815	388 (47.6%)	42		35	49

^a^ 0.95 LCL: 95% Lower Confidence limit. ^b^ 0.95 UCL: 95% Upper Confidence limit

### SAM in taller children are easily captured by WHZ

The community survey data shows that the children with tall height who fall on upper quartile in Height for Age (HFA) index have (are exposed to) significantly higher risks for SAM by WHZ compared to those with short stature whose height falls on lower quartile (Odd Ratio, OR: 1.63, *p* = 0.049). The OR increases for the children with SAM-WHZ who are normal by MUAC (OR: 2.24, *p* = 0.006, Table 2. C). However, the OR for SAM by MUAC is higher for those children who fall on the lower quartile (OR: 0.42, *p* = 0.0001). This result proves that the children with tall height are likely to be classified with acute malnutrition by WHZ but normal by MUAC.

### MUAC is more linked to HAZ, or severe stunting, and comprises traits of chronic malnutrition

The Venn diagram using the survey data indicates that the prevalence of severe stunting among the children with SAM by MUAC is greater than that among children with SAM by WHZ (12.2%vs. 7.5%). This is also consistent for those with GAM (11.7% vs. 6.3%) (Fig. 2). The Spearman’s correlation coefficient between MUAC and HAZ is greater than that between WHZ and HAZ (0.182, *p* =2.2 × 10^−7^ vs. -0.100, p =6.405 × 10^−9^) from the community survey data. The *χ*^2^ statistics based on a 2 × 2 contingency table (Table 2. D)between SAM-MUAC (MUAC< 115, MUAC≥115) and severe stunting (HAZ < − 3, HAZ ≥ − 3) is also larger than that between SAM-WHZ (WHZ < -3, WHZ ≥ -3) and severe stunting (27.9053, p = 1.274 × 10^−7^ vs. 0.5517, *p* = 0.4576). Accordingly, the odds ratio estimate from the 2 × 2 contingency table between SAM-MUAC and severe stunting is also larger than that between SAM-WHZ and severe stunting: 3.38 with the 95% confidence interval (3.03, 3.77) vs. 1.33 with the 95% confidence interval (1.10, 1.60).

For the program data, after where the data of healthy children are inevitably excluded, the correlation coefficient is estimated by the likelihood approach using the truncated regression model [41]. This method enables the estimation of the coefficient regardless of the type of distribution of the population. The analysis showed a similar result displaying higher correlations between SAM-MUAC and moderate stunting (HAZ < -2) than SAM-WHZ and moderate stunting (HAZ < -2) (0.45 vs.-0.07). The results consistently demonstrate that SAM-MUAC is more closely related to chronic malnutrition.

### Adjusted MUAC screening cut-off values to represent SAM-WHZ in South Sudan children

**Fig. 3:**
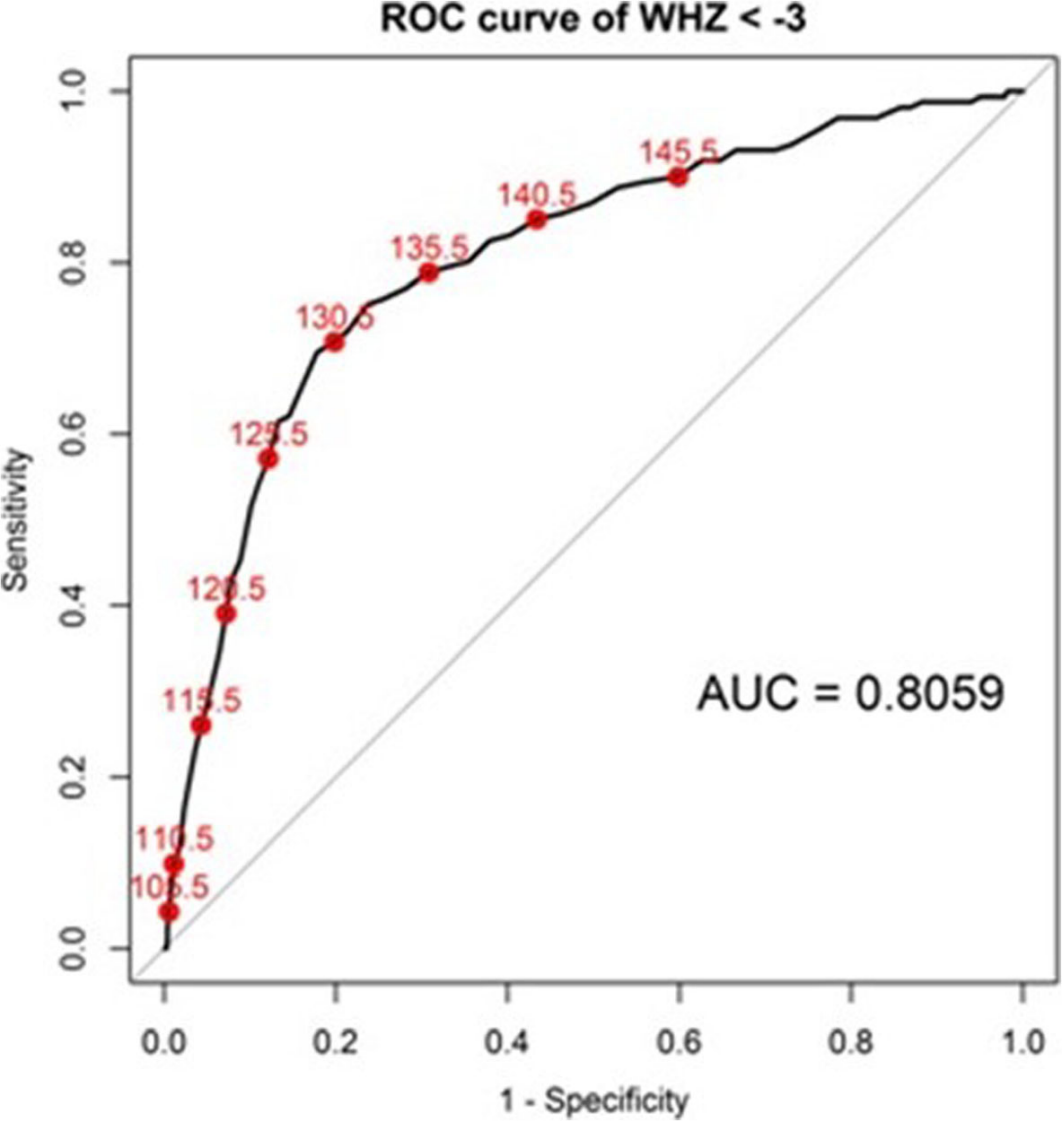
The ROC curves for predicting SAM-WHZ by MUAC. AUC, Area Under Curve; ROC, Receiver Operating Characteristic

The ROC curve of MUAC to identify SAM-WHZ is traced for the cut-offs. The MUAC cut-off value for the SAM-WHZ is estimated by Youden index (Sensitivity + Specificity – 1) resulting in 129 mm for SAM with sensitivity and specificity 69.6 and 82.2%, respectively, while 135 mm for GAM-WHZ with sensitivity and specificity 68.5 and 74.5%, respectively (Fig. 3). If 133 mm is taken for a cut-off point for the SAM by WHZ as suggested by Laillou [17] sensitivity and specificity will reach 75.8 and 70.8%, respectively.

### Kaplan-Meier survival analysis (Table 2. E)

Q1, Q2, and Q3 of MUAC and WHZ at admission for the program was (11, 11.5, and 12.2) and (− 3.975, − 3.104, and − 2.126), respectively. The Kaplan-Meier survival analysis of the program data showed that the median times for recovery to 15% weight gain are similar for SAM’s by WHZ and MUAC (Table 2. E.①) in the sense that the 95% confidence intervals are overlapping [42]. Similar results were found for the survival analysis on the recovery times for the 5% MUAC gain (Table 2. E.②).

### Comparison of relative increase rate of MUAC^3^ and weight during nutrition program

**Fig. 4:**
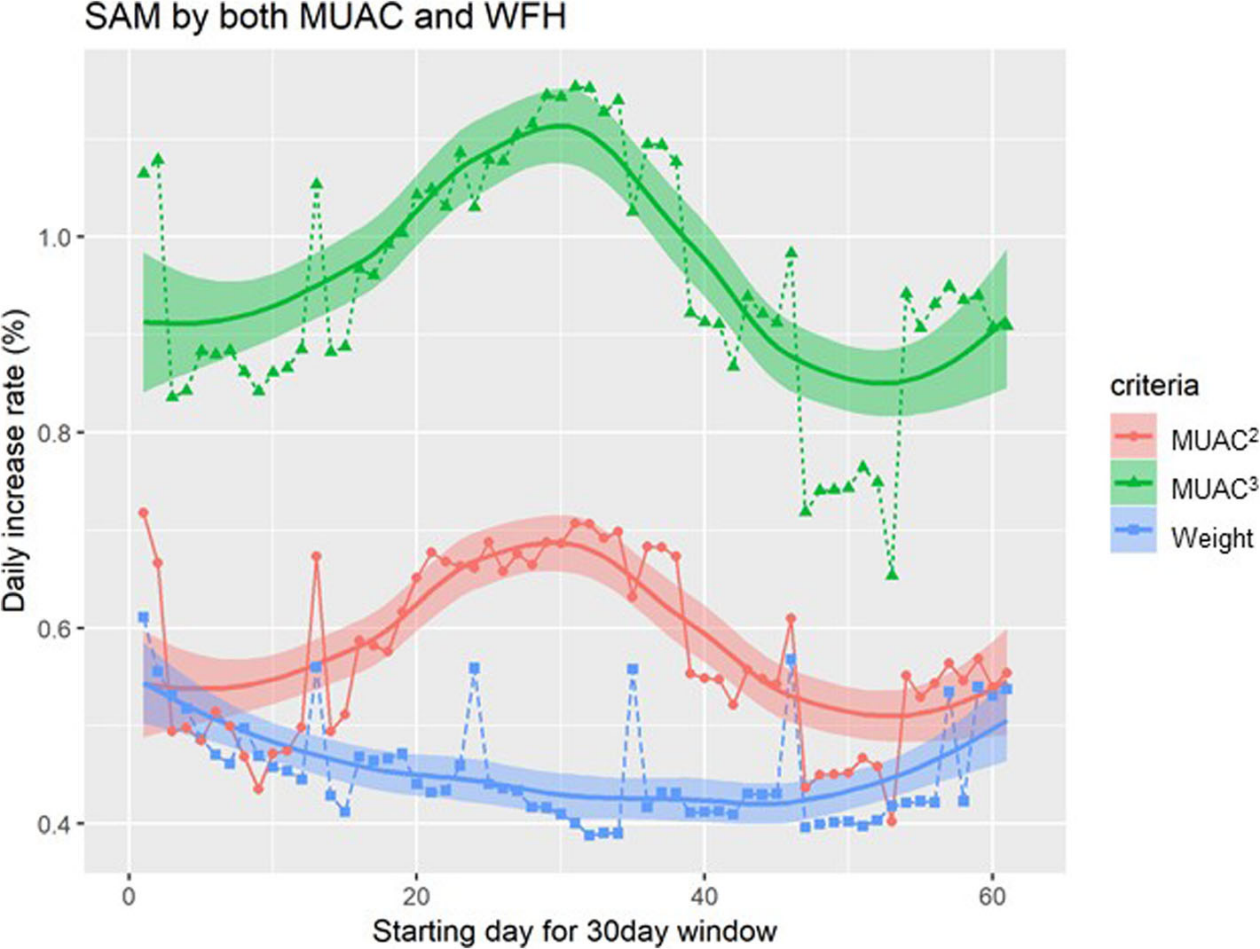
Daily increase rate during recovery period. Average daily relative increase rates (%, based on initial admitted measurements) of MUAC^2^ (red), MUAC^3^ (green), and Weight (blue) are calculated using a sliding window method (30 day width). Locally Weighted Scatterplot Smoothing (LOWESS) is used to smoothen sliding window results

Among the children with SAM both by MUAC and WHZ, the relative rate of MUAC^3^ gain is significantly faster than that of weight gain all over the recovery period until discharge. Both MUAC^3^ and weight gain show initial and terminal peaks but MUAC^3^ has another bigger accelerated phase between the peaks while weight gain become plateau after the first peak and regain its velocity after 40 days of admission. (Fig. 4). The average daily MUAC^3^ gain calculated as above in SAM-MUAC and SAM-WHZ (0.33% vs 0.27%, *p*-value = 0.054) was more distinct compared to average daily weight gain (0.77% vs 0.60%, *p*-value < 0.001), respectively. And we carefully suggest that fat mass restoration tend to be predominant especially during the period between 15 and 50 days. We also compared average daily gain of MUAC^3^ and Weight in each of the two SAM groups separately. To clearly observe the recovery after the treatment, we only analyse the data from children with records between 30 days to 60 days and calculate the average daily increase from the first visit till the last day before 60 days. For example, if a child came one day and 9, 20, 32, 45, 56, and 72 days after this child revisit the center to receive packets of food, the child’s physical measurements are recorded at each visit. Then, average daily increase of the weight or MUAC is calculated based on the difference between the first visit and 56 day visit.

## Discussion

As demonstrated in other series of studies [14, 16], our data also show poor correspondence between the two SAM’s (GAM’s) identified by MUAC and WHZ, displaying only 12.3% (26.5%) are overlapped. Similar to other studies [6, 17, 43], our study also shows that children with tall height are prone to be classified as malnourished by WHZ but normal by MUAC. This finding might suggest that some children with tall height are prone to be classified as malnourished by WHZ, while they do not have actual malnutrition. Even after exclusion of those children with SAM-WHZ with tall height, but normal by MUAC, the correlation estimation between each type of acute malnutrition and HAZ still showed higher between MUAC and HAZ than between WHZ and HAZ (0.185 vs. -0.096). This procedure prevents a biased decrease in correlation between acute malnutrition by WHZ and stunting, and leads to biased relative increase in correlation between MUAC and stunting. Besides our community survey data also demonstrate that MUAC identifies younger children and more girls as malnourished than WHZ, which is similar to observations in other studies [8, 44].

The community survey data show that the malnourished children by SAM-MUAC are more severely stunted (HAZ < − 3) than those by SAM-WHZ. Considering that the children with severe stunting would have the least chance to reach their genetic height even after the treatment, SAM-MUAC would not be a good screening modality to identify malnourished children for enrolment in a management program to prevent short stature. Here, we found that major portion of included children by low MUAC are already stunted and more loose criteria based on MUAC is required to capture a group of children with potential high risk in stunting. The observed correlation coefficient between MUAC and HAZ from community survey data is larger than that between WHZ and HAZ. Additional 2 × 2 contingency table analyses showed that there is a strong statistically significant association between SAM-MUAC and chronic malnutrition (HAZ < -3) (*p* = 1.274 × 10^−7^), but no statistically significant association was found between SAM-WHZ and chronic malnutrition (HAZ < -3) (*p* = 0.4576). This tendency persists even after exclusion of the tall children with SAM-WHZ but normal by MUAC. Together with the data from hospitalized children in Kenya showing that the children with SAM-MUAC tended to have a more chronic clinical feature (Kwashiorkor) than those by WHZ (5.0% vs. 49.0%, *p* < 0.001) [44], our finding supports that MUAC screening identifies more advanced malnourished cases than the screening by WHZ. Additionally, if children were with edema, there was strong geographical variations in their MUAC [45]. Thus, we might reconsider using the term of ‘acute’ for those malnourished cases identified by MUAC.

In early stage of wasting when a reasonable amount of fat in the body is retained, MUAC represents fat mass of the children [46]. In starvation, the body starts to use fat for energy source soon after the body faces insufficient energy intake [47]. However, body muscle mass is maintained at the later stage of malnutrition since protein catabolism is maintained at a minimum and the organism lives on fat body stores [48]. The median duration to progress to SAM is estimated about 7.5 month [49]. During this progression, the body mainly utilises fat for the energy source to support life. Therefore, in severely malnourished children with limited fat mass, the arm circumference represents remaining body muscle mass instead of fat [50]. Additionally, in severely malnourished children, infection is frequently super imposed [51–55], when the reserved muscle mass is further to be utilized for amino acids resources for immune response as well as for energy resources [56].

Though WHZ screening identifies earlier malnutrition cases, it also has a limitation to identify normal children with tall height without malnutrition especially for the ethnic groups with long limbs as evidenced in this study.

The data also showed the relative velocity MUAC^3^ gain surpassed that of weight gain among the children with SAM both by WHZ and MUAC over the recovery period. Based on our data analysis and simple idea that fat has lower density than other body components, we suggest fat mass gain was faster than that of lean body mass during recovery period in South Sudan nutrition program. Also this conjecture is duplicated with the previous observation among severely malnourished children who were undergone parenteral nutrition for management [57]. Besides, considering that bone growth reflecting limb length does not occur simultaneously with weight and fat mass gain, MUAC^3^ gain could exaggerate real volume increase. However, the relative velocity of MUAC^2^ gain consistently surpassed that of weight gain but in a lessor scale than MUAC^3^ gain (Fig. 4) with similar velocity peaks as in MUAC^3^ gain. Therefore, if mortality is related to decreased muscle mass [58], it is recommended to provide care for the malnourished until full muscle mass restoration even after the malnourished children has reached discharge criteria.

## Conclusion

MUAC does not simply identify acute stage of malnutrition but also identifies more advanced malnourished cases than WHZ among the children in the South Sudan community. Our result supports that each screening modality by MUAC or WHZ identifies not only different characteristics but also different stages of malnutrition. Therefore, each screening modality should be used as a complementary to each other, as MUAC and WHZ identify different malnourished cases. Considering that the goal of community screening is to identify malnourished children in a timely manner and prevent not only mortality but also chronic complications including short stature, it is advised to employ both WHZ and MUAC measures at the community for acute malnutrition screening. Specifically, when MUAC is the only measure for the screening, it is recommended to use more loose MUAC cut-off value (133 mm) than current 125 mm to avoid excluding children who could be admitted by SAM-WHZ. This study also suggests initial weight gain is mainly ascribed to fat mass increase among the children with SAM, the current discharge criteria, restoration of anthropometric data still leave the malnourished children with insufficient restoration of muscle mass, and at risk of mortality after discharge. Given this, further research should be followed including assessment of body composition to revise the current admission and discharge criteria for CMAM program.

## Data Availability

The datasets used and/or analysed during the current study are available from the corresponding author on reasonable request.

## Abbreviations

CMAM: Community Management of Acute Malnutrition
HFA: Height For Age
HAZ: Height For Age Z score
GAM: Global Acute Malnutrition, defined as MUAC < 125 mm or WHZ < -2
SAM: Severe Acute Malnutrition, defined as MUAC < 115 mm or WHZ < -3
MUAC: Mid Upper Arm Circumferences
SAM-MUAC: Severe Acute Malnutrition identified by MUAC
SAM-WHZ: Sever Acute Malnutrition identified by WHZ
UNICEF: United Nations Children’s Fund
WHO: World Health Organization
WFA: Weight For Age
WFH: Weight For Height
WHZ: Weight for Height Z score
